# Platelet Rich plasma injection of the vocal folds in benign vocal pathologies

**DOI:** 10.1007/s00405-024-08824-5

**Published:** 2024-07-17

**Authors:** Georgia Mackay, Jacqui Allen

**Affiliations:** Department of Surgery, University of Auckland, Private Bag 91019, Grafton Auckland, New Zealand

**Keywords:** Platelet rich plasma, Dysphonia, Vocal fold, Scarring, Atrophy, Sulcus

## Abstract

**Purpose:**

There are few options for treatment of dysphonia secondary to vocal pathology related to lamina propria scar, atrophy, sulcus, or inflammatory disorders. Platelet rich plasma (PRP) may provide anti-inflammatory and regenerative properties seen with other tissue engineering therapies without the risks associated with these treatments. We evaluated vocal fold (VF) injection of PRP for feasibility, phonatory effects, patient satisfaction and durability.

**Methods:**

Patients with dysphonia secondary to vocal fold scar, atrophy, sulcus and inflammatory lesions were included. PRP injections were administered in office, to bilateral vocal folds. Patients were followed up at 1 week, 1 month, 3 months and 6 months to assess outcomes (GRBAS scale, maximum phonation time, vocal fatigue index (VFI), voice handicap index (VHI-10) and stroboscopy).

**Results:**

75 intracordal PRP injections were administered to 48 patients. All injections were completed, and no adverse reactions were experienced. Improvements in VHI-10 scores at 1,3,6 months were seen (mean VHI 21.73 at baseline, 15.62 at six months, *p* < 0.001). 72.3% rated improvement at 7 or above on Likert scale. 95.7% of patients would consider a future PRP injection. Secondary outcomes VFI, MPT, and GRBAS also demonstrated significant improvements over time. Patients receiving a single PRP injection (*n* = 26) still demonstrated significant VHI-10 improvements at 1,3 and 6 months.

**Conclusions:**

VF office PRP injections are feasible and safe and can provide phonatory benefit and reduce vocal effort in benign VF disorders. A single PRP injection is sufficient to provide sustained benefit in some cases.

**Level of evidence:**

Level III: prospective cohort study.

## Introduction

The intricate microcellular environment of the vocal fold (VF) is intimately related to vocal function [1]. Precise ratios of collagen, elastin, and hyaluronic acid within the superficial lamina propria (SLP) of the VF contribute the viscoelastic and strength properties that allow for VF oscillation and sound production [2–4]. Unlike the skin, where scars and defects merely appear unsightly, scarring of the VF significantly impairs vocal function [5, 6]. Even small disruptions to the unique architecture of the VF can cause significant dysphonia, due to impairments of wave oscillation, or VF free edge irregularities hindering full glottic contact. VF scar, sulcus and atrophy sit along a spectrum of disorders affecting the LP of the vocal fold [7–11]. Whilst their aetiologies and pathogenesis differ, they share similar features of inflammatory change, collagen dysregulation, and loss of the glycosaminoglycans and hyaluronic acid [2, 12, 13]. Unlike VF paralysis (where medialisation of the affected VF into the airflow stream can allow for normal glottic contact and VF oscillation), using medialisation techniques for VF scar, sulcus and atrophy yield underwhelming results, as glottic insufficiency is only one element of the problem [14]. Similarly, the placement of exogenous substrates into the VF can result in poor vocal outcomes due to differing vibratory properties of manufactured substances and substance mass compared to native VF SLP components [15, 16].

Research into biological techniques have gained popularity in laryngology for their proposed ability to stimulate regeneration of the lamina propria. The current biologics are in various stages of in vitro, animal and human testing, and include recombinant growth factors (bFGF and HGF) [12, 13], and stem cell treatments derived from bone marrow and adipose tissue [17–19]. Whilst promising results have been shown in support of the regenerative properties of these techniques, they are resource intensive, expensive, and the ethical and regulatory protocols surrounding this field frequently hinder their progression to widespread human application [6].

Platelet rich plasma (PRP) whilst new in laryngology, is a common treatment option in many fields of medicine, including rheumatology, appearance medicine and plastic surgery [20–23]. Like growth factors and stem cells treatments, PRP is hypothesised to stimulate healing processes in tissues with low healing potentials, and reverse fibrotic processes [24–26]. The growth factors, cytokines and anti-inflammatory properties of PRP provide the theoretical basis for its use in the VFs (PDGF, VEGF, HGF, bFGF) [27–31]. PRP has been shown to reduce NF-kB mediated inflammation [26, 27, 29], and reduce expression of COX-1, COX-2 inflammatory mediators [30]. PRP has been shown to revert TGF-b1 mediated fibrotic and inflammatory signalling pathways [24, 28]. Many methods of PRP preparation exist, and in general they are quick, cost-effective and require minimal training [32–34]. Unlike expensive implants and current commercially-manufactured injection substrates, the cost of PRP is small, limited to standard blood draw equipment, use of a centrifuge machine, which can be found in most centres with lab facilities. There is no direct implant cost as the PRP comes from the patient. Animal and human studies of PRP injections into the VF have confirmed its safety, with no reports of inflammatory reactions, scarring, granulomas or other significant adverse outcomes [31, 35–42]. Current human studies of the use of PRP in the VFs, show improvement in phonatory outcomes out to 1 year after PRP injections, with small cohort sizes and variable follow up duration, meaning further assessment is needed to guide regular office-based use [38, 39, 42].

This study aims to evaluate safety and phonatory outcomes of intracordal PRP injection for benign VF pathology.

## Materials and methods

Ethical consent was obtained for this study from the Health and Disability Committee of New Zealand (HDEC Central committee: 2022 FULL 13,929). Patients were treated with bilateral intracordal PRP injections in all cases.

### Inclusion and exclusion criteria

Patients were included if they were over 18 years old and reported dysphonia secondary to benign VF disease including: VF scar, atrophy, sulcus and inflammatory lesions. Patients were excluded if a progressive neurological condition was the cause of their dysphonia, malignancy involved the larynx, or they demonstrated clotting disorders or were pregnant. Alternative treatments were offered to patients, and they were given time to consider consenting to PRP injections. Patients with voice change of all severity levels were included, and patients who had received prior treatment with other modalities were eligible for inclusion. As the study aim was to assess safety, tolerance and patient-reported voice benefits, we included individuals with different aetiologies of voice change. This study was not blinded or randomized. All participants received PRP injection.

### PRP preparation

Two 8.5 ml samples of blood were drawn into acid citrate dextrose (ACD) vacutainers [Capes Medical, Mangere, New Zealand] via standard phlebotomy technique from the antecubital fossa (ACF) or dorsum of the hand for each patient. Blood samples were centrifuged at 3500-4000 rpm (2030RCF) for 10 min using a Frontier™ 5000 Series Multi-Pro OHAUS centrifuge (FC5714). After centrifugation, the top 2/3 of plasma from each sample was discarded using a 10 ml syringe with a blunt 18-gauge needle. The remaining 1/3 of plasma above the buffy coat (the PRP supernatant) was extracted with a 1 ml syringe, with careful precision so as not to include any red blood cells in the sample.

### PRP injection

Patients received PRP injections via transnasal or transcervical (endoscope-guided thyrohyoid approach) standard techniques under local anaesthetic in the office, into the superficial lamina propria. All patients were given bilateral PRP injections regardless of unilateral or bilateral pathology. VFs were over injected until grossly volumized (usually with a yellow coloration), or until spontaneous supernatant extrusion occurred, typically between 0.25 and 0.8 ml each side.

Patients were monitored after injections for 15 min for immediate reactions. Patients were recommended 24 h of voice rest immediately following the procedure to ensure maximal absorption of PRP.

### Assessment of voice outcomes

At initial appointment patients completed voice handicap index (VHI-10) and vocal fatigue index (VFI) tools and then during videostroboscopic assessment, performed a range of voiced tasks including range, maximum phonation time (MPT) and whisper speech. The clinician rated the voice using GRBAS scale (grade, roughness, breathiness, asthenia, strain) and estimated mucosal wave amplitude and glottal closure (complete vs. incomplete). All evaluations were performed at each follow up appointment (1, 3, 6 and 12 months) after injection, alongside stroboscopy. Stroboscopy assessment included visualisation of mucosal wave form, glottic contact, phase symmetry and amplitude excursion. Furthermore, at post-injection appointments, patients were asked to rate on a Likert scale (1–10) their willingness to undergo future PRP injections, and their perceived degree of voice improvement.

### Statistical tests

Paired t-tests were used to compare pre-injection and post-injection outcomes for parametric data, and Wilcoxon Signed Rank test for non-parametric data. Independent t-tests were used to compare degree of VHI-10 improvement by pathology. Pearson’s correlations were used to compare degree of VHI-10 improvement and age. P value of < 0.05 was considered significant. All statistical tests were performed using SPSS 23 (SPSS, IBM, USA).

## Results

A total of 75 intracordal PRP injections were administered to 48 patients over the one-year study period. There were 28 patients with VF atrophy (58.3%), 10 with sulcus or scar (20.8%), and 10 with inflammatory VF disease (20.8%). The study group consisted of 16 males (33.3%), and 32 females (66.7%), with an ethnicity distribution of 38 (79.2%), 3 (6.3%), 3 (6.3%) and 1 (2.1%) for New Zealand European, Asian, Māori and other respectively, 3 patients (6.3%) did not report ethnicity. The ages ranged from 18 to 82 years, median 62 years old (mean 56 years, standard deviation 18.1). 21/48 patients had received previous VF procedures including injection laryngoplasty, Gore-tex™ implants and laser treatment but not within the preceding six months prior to PRP injection. No patient withdrew from the study; however one moved overseas and was lost to follow up, and two patients were excluded from further follow up after receiving other vocal fold procedures. 47 patients had available follow up data at the completion of the study. 26 (54%) patients underwent a single injection, and 22 (46%) patients received 2 or more injections.

Twenty-six patients (54.2%) were professional voice users. The most common voice presenting symptom reported was reduced volume in 23 patients (47.9%), followed by vocal fatigue (12 patients, 25.0%), poor sound quality (9 patients, 18.8%) and odynophonia (4 patients, 8.3%). Mean duration of vocal symptoms was 4.2 years (standard deviation 4.3), (range 0.5–25 years).

All patients received bilateral injections. Average injectate volume on each side was 0.38 ml (standard deviation 0.09, range 0.1-0.7 ml). 43 injections (57.3%) were administered via a transcervical route, and 32 injections (42.7%) by transnasal approach.

### Safety

No patient had any significant adverse reaction to PRP injection. The procedure was generally well tolerated with mild discomfort for up to 48 h after injection reported by 17/46 participants (37.0%).

### Patient reports

**Fig. 1:**
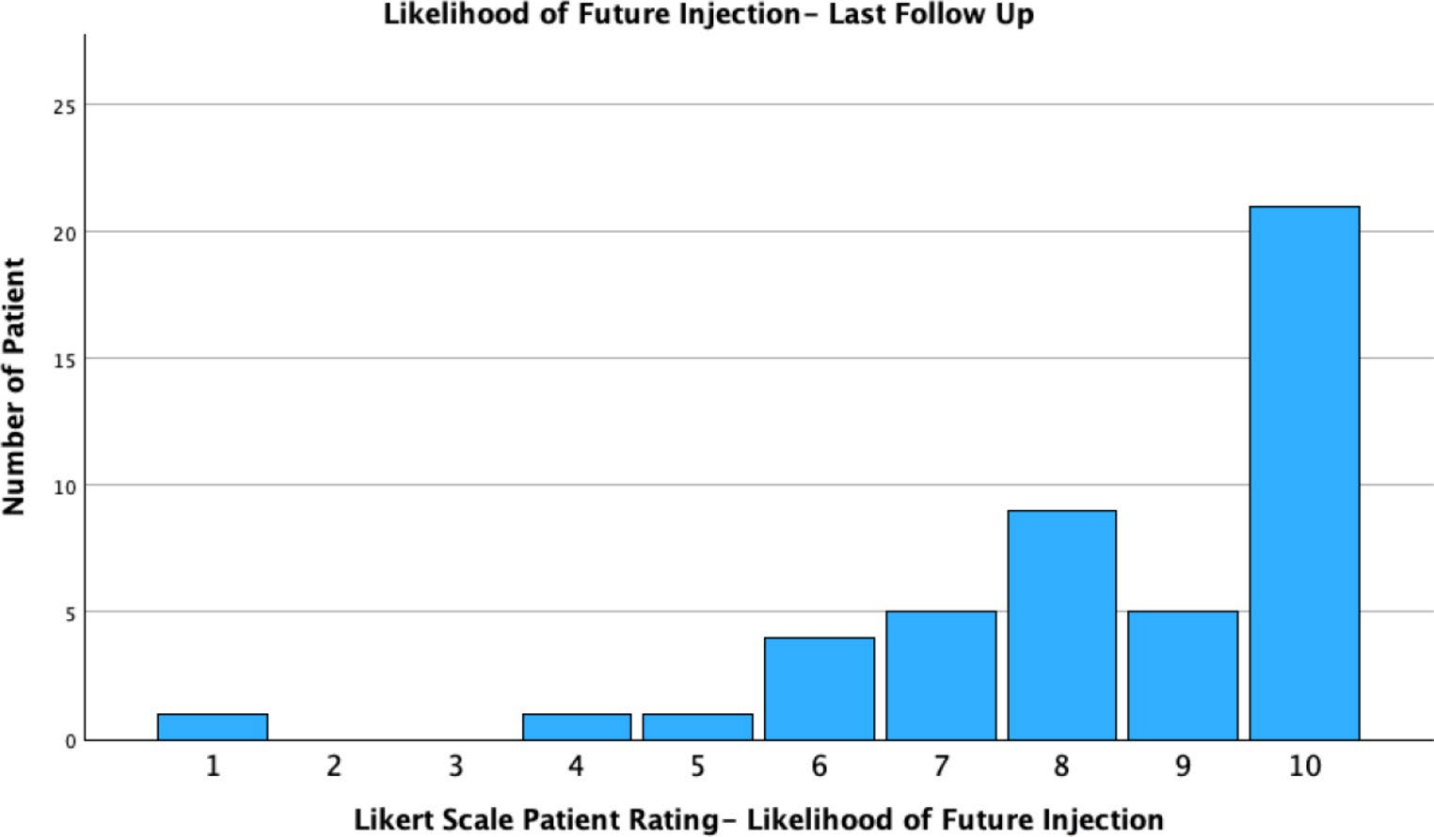
Likert scale (1–10) measures for question “How likely are you to consider a future PRP injection?” at last patient follow up

**Fig. 2:**
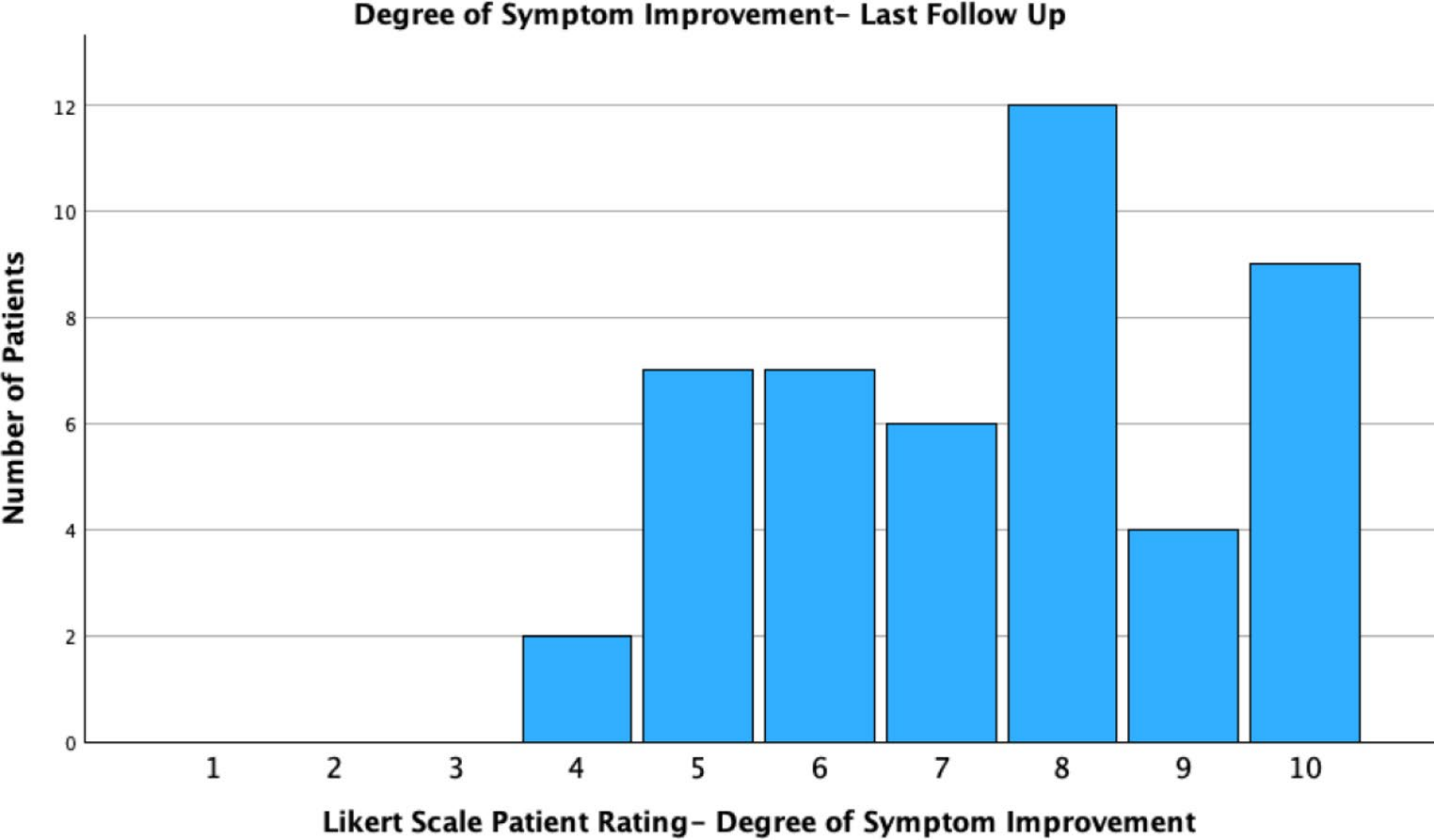
Likert scale (1–10) measures for question “How would you rate your degree of vocal improvement after PRP injection?”

Participants were asked to rate degree of symptom improvement and their willingness to undergo a future PRP injection on a Likert scale from 1 to 10. Forty seven patients had Likert data available, 72.3% (34/47 of patients) rated their improvement at 7 or greater on the Likert scale during follow up. 45/47 (95.7%) reported they would consider having a future PRP injection at their last follow up (≥5 Likert rating). Two participants expressed reluctance towards future injections after experiencing procedural discomfort during the injection, but would consider if performed under a general anaesthetic (Figs. 1 and 2).

### Outcomes

**Fig. 3:**
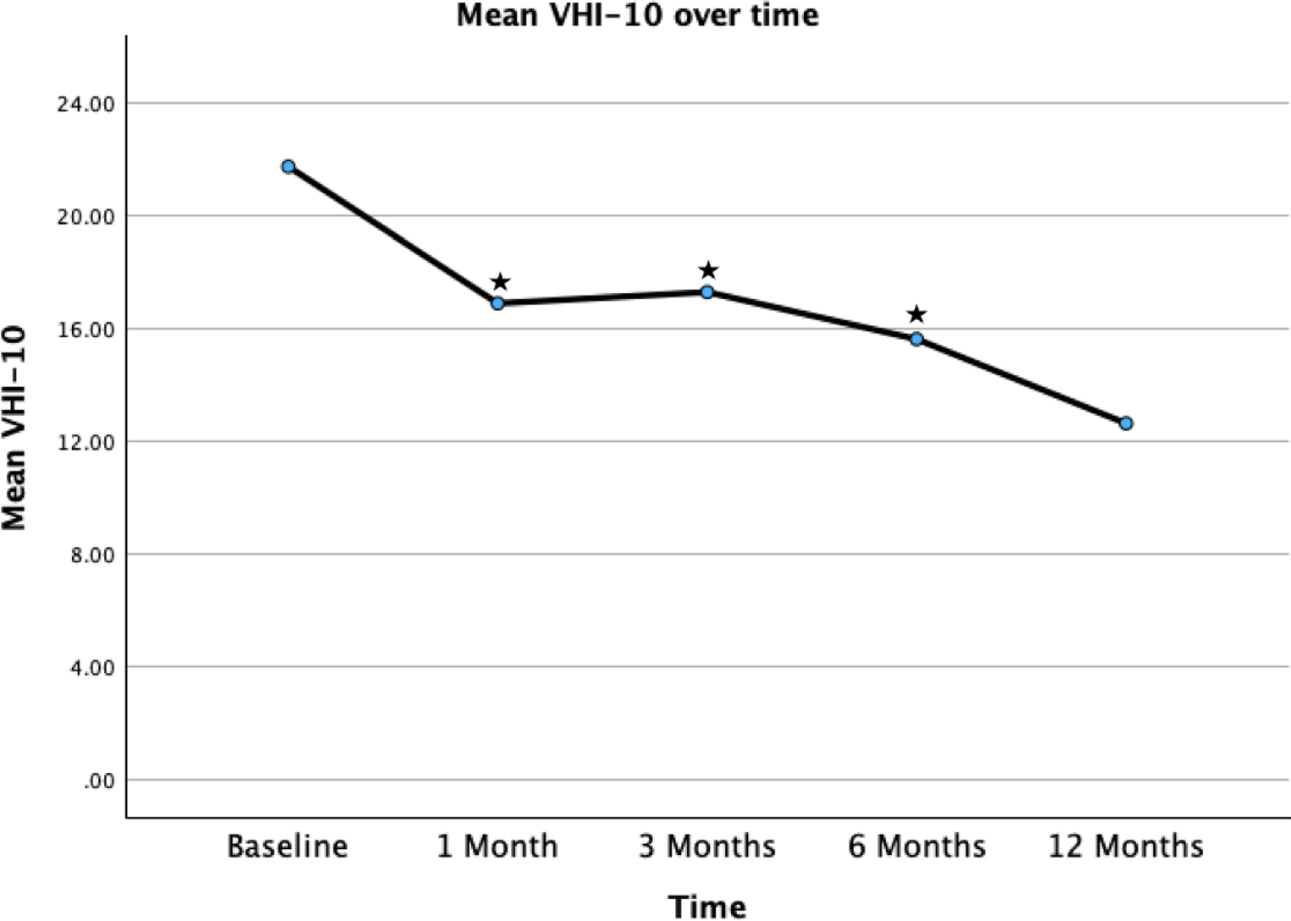
Mean VHI-10 scores over time. Star represents significant change compared to baseline values on paired t-test. ^*^ Represents statically significant comparisons (two tailed p value < 0.05) with baseline value

Table 1 displays the pair-wise comparisons of the primary study outcomes (VHI-10, VFI, MPT) at various follow up time points, with all results compared to baseline values. Figure 3 displays improvements in VHI-10 across time. 32/47 patients (68.1%) achieved the minimum clinically important difference (MCID) of a decrease in VHI-10 equal to or greater than 5 during the follow up period. There was a significant difference between the baseline mean VHI-10 score and the 1-, 3- and 6-month scores.

**Table 1:** Comparison of mean values for VHI-10, VFI parts 1 and 2 and MPT across study time points

	Baseline	1 month	3 months	6 months	12 months
VHI-10	**21.73** (*n* = 45)	**16.89** (*n* = 45)*p < 0.001	**17.29** (*n* = 35)*p = 0.006	**15.62** (*n* = 29)*p < 0.001	**12.63** (*n* = 8) *p* = 0.115
VFI-1	**28.05** (*n* = 41)	**22.70** (*n* = 43)* *p* < 0.001	**20.82** (*n* = 34)* *p* < 0.001	**18.46** (*n* = 26)*p < 0.001	**13.50** (*n* = 6)* *p* = 0.004
VFI-2	**7.53** (*n* = 43)	**6.35** (*n* = 43)*p = 0.047	**4.94** (*n* = 34)*p = 0.002	**4.77** (*n* = 26)* *p* < 0.001	**2.17** (*n* = 6) *p* = 0.16
MPT	**14.49** (*n* = 43)	**17.67** (*n* = 43)* *p* < 0.001	**16.19** (*n* = 32)* *p* = 0.001	**15.59** (*n* = 22) *p* = 0.101	**20.20** (*n* = 6)* *p* = 0.022

* Represents statically significant comparisons (two tailed p value < 0.05) with baseline value

There were significant improvements in VFI1 scores at all time points, and improvements in VFI2 scores at 1, 3 and 6 months (Table 1). There was no significant improvement in VFI3 at any time point.

**Fig. 4:**
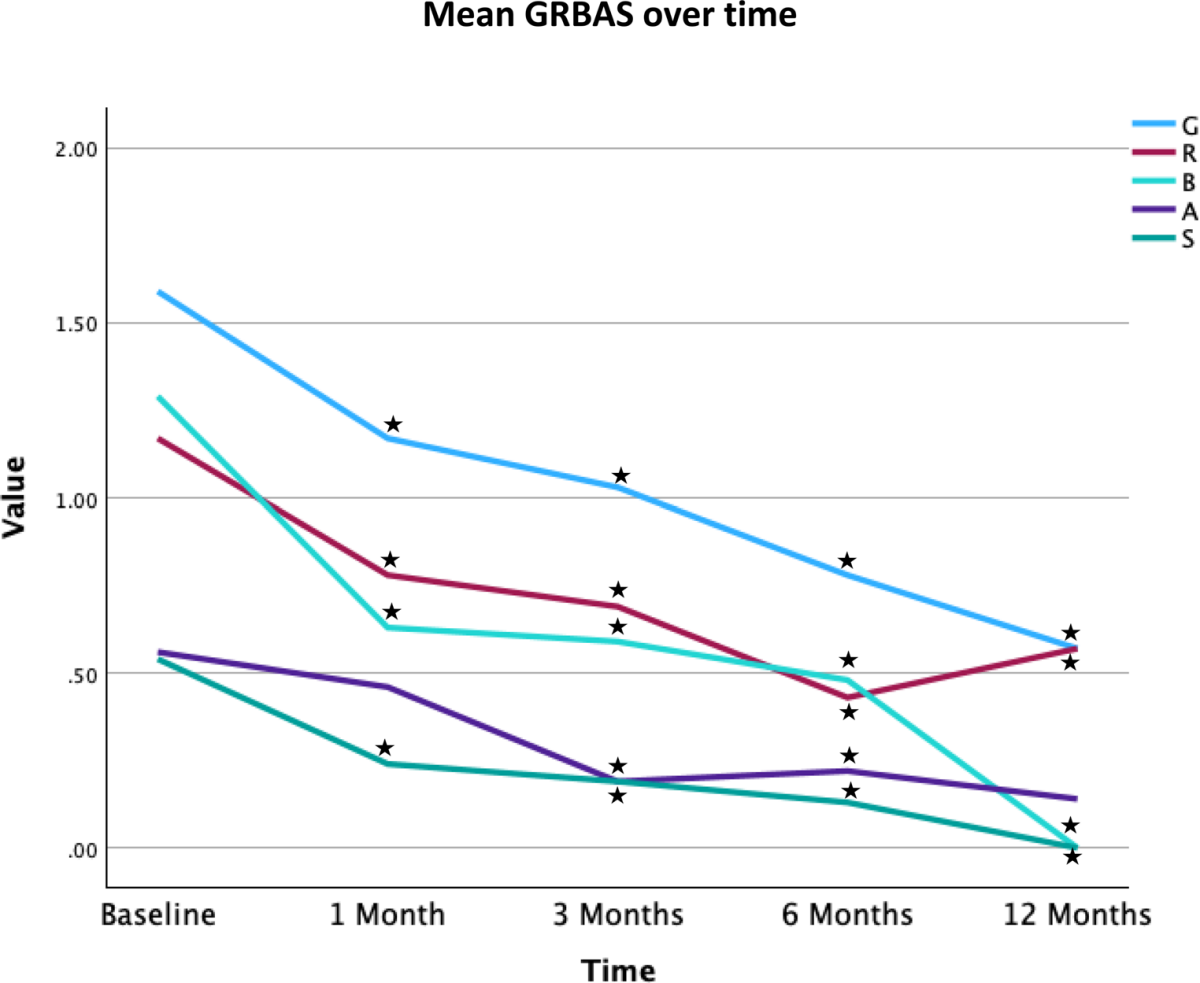
Mean GRBAS ratings over time. Star represents significant change compared to baseline values on paired t-test. ^*^ Represents statically significant comparisons (two tailed p value < 0.05) with baseline value

Figure 4 display mean GRBAS measurements over time. Paired t-tests yielded significant reduction in scores at all time points for G, R, B, S compared to baseline. Aesthenia ratings showed significant reduction at 3 and 6 months only.

**Fig. 5:**
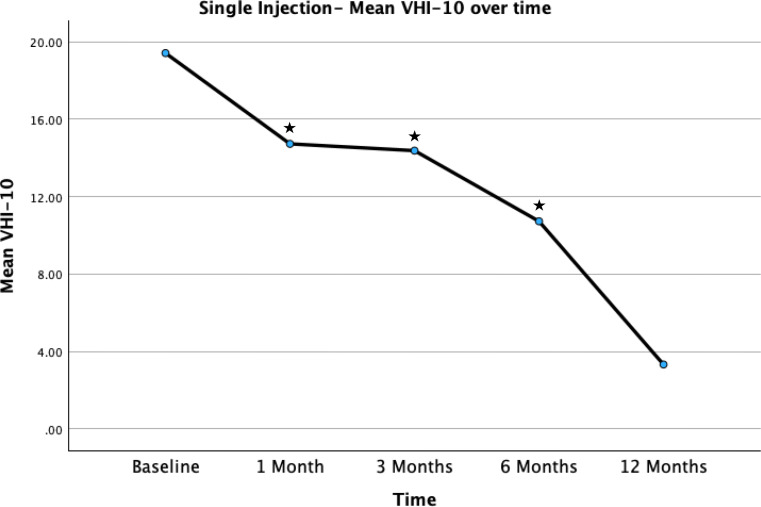
Mean VHI-10 scores over time for patients with single injections. Star represents significant change compared to baseline values on paired t-test. ^*^ Represents statically significant comparisons (two tailed p value < 0.05) with baseline value

***Subgroup analysis*** was undertaken of patients who underwent a single injection (*n* = 26) only. There was significant improvement in mean VHI-10 comparing baseline VHI-10 (19.42) to 1 month (14.73) (*p* < 0.001) (*n* = 26), 3 months (14.38) (*p* = 0.015) (*n* = 13) and 6 months (10.73) (p = < 0.001) (*n* = 11), displayed in Fig. 5.

There was no significant difference in mean VHI-10 change between groups related to VF pathology or age.

When analysed by aetiology, the atrophy (*n* = 26) group saw significant VHI-10 improvements at 1 month (mean change VHI-10 5.6 SD 7.7, *p* = 0.001), 3 months (mean change VHI-10 3.7 SD 7.2 *p* = 0.035) and 6 months (mean change VHI-10 6.1, SD 6.3, *p* = 0.002) compared to baseline, and there were also significant improvements for VFI1, and VFI2 at 1,3 and 6 months in the atrophy group. Scar/sulcus patients (*n* = 10) did not demonstrate significant VHI-10 improvements across any time period. The inflammatory group (*n* = 10) demonstrated significant VHI-10 improvement at 1 (mean change VHI-10 6.0 SD 8.0 *p* = 0.042) and 3 months (mean change VHI-10 8.8 SD 3.7 *p* = 0.002).

### Stroboscopy

**Fig. 6:**
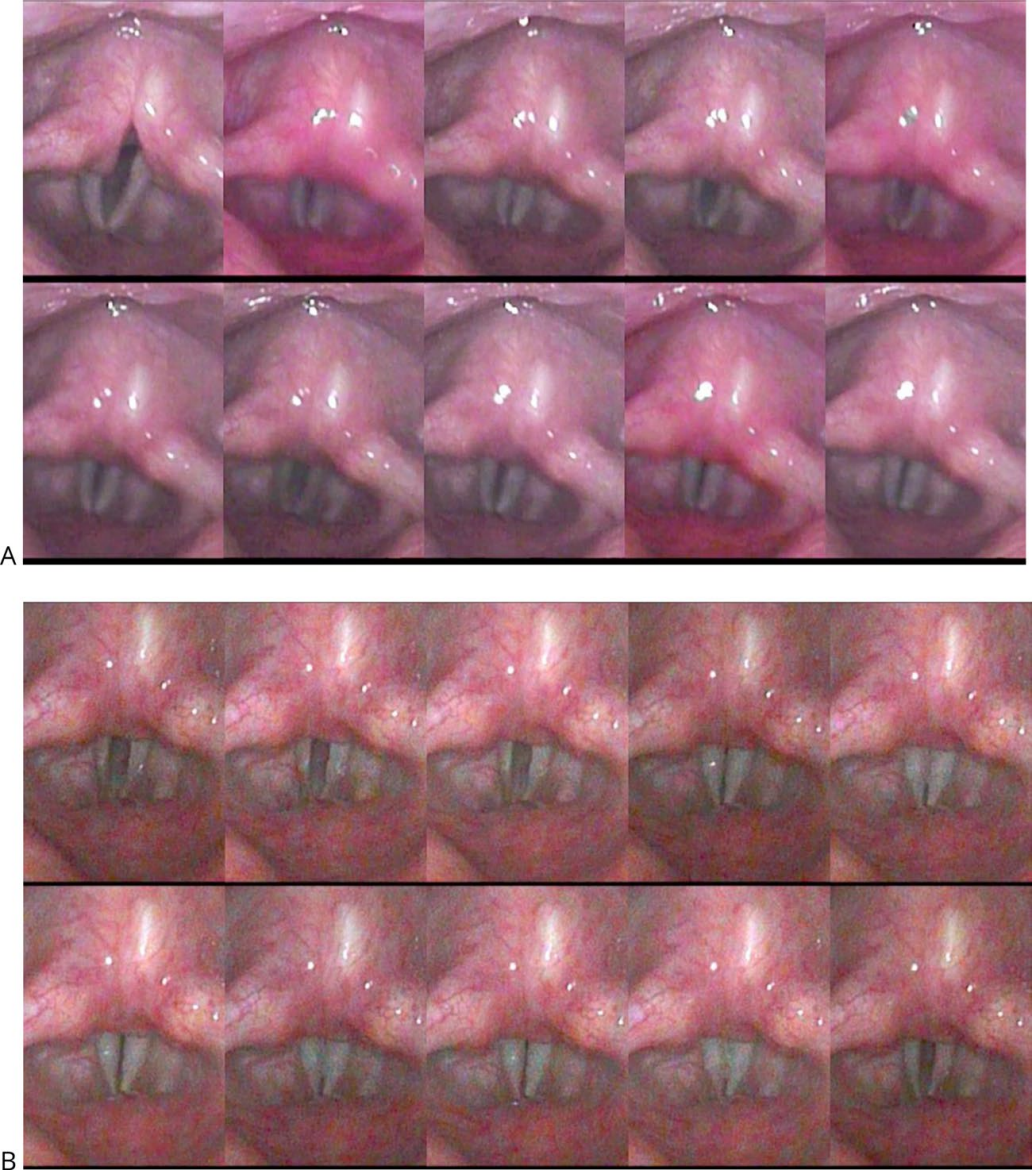
Stroboscopic image montage of representative case. **A**) pre-PRP injection demonstrating glottic gap throughout glottal cycle, and **B**) one month after PRP injection demonstrating reduced glottal gap and increased closed phase of glottic cycle

**Fig. 7:**
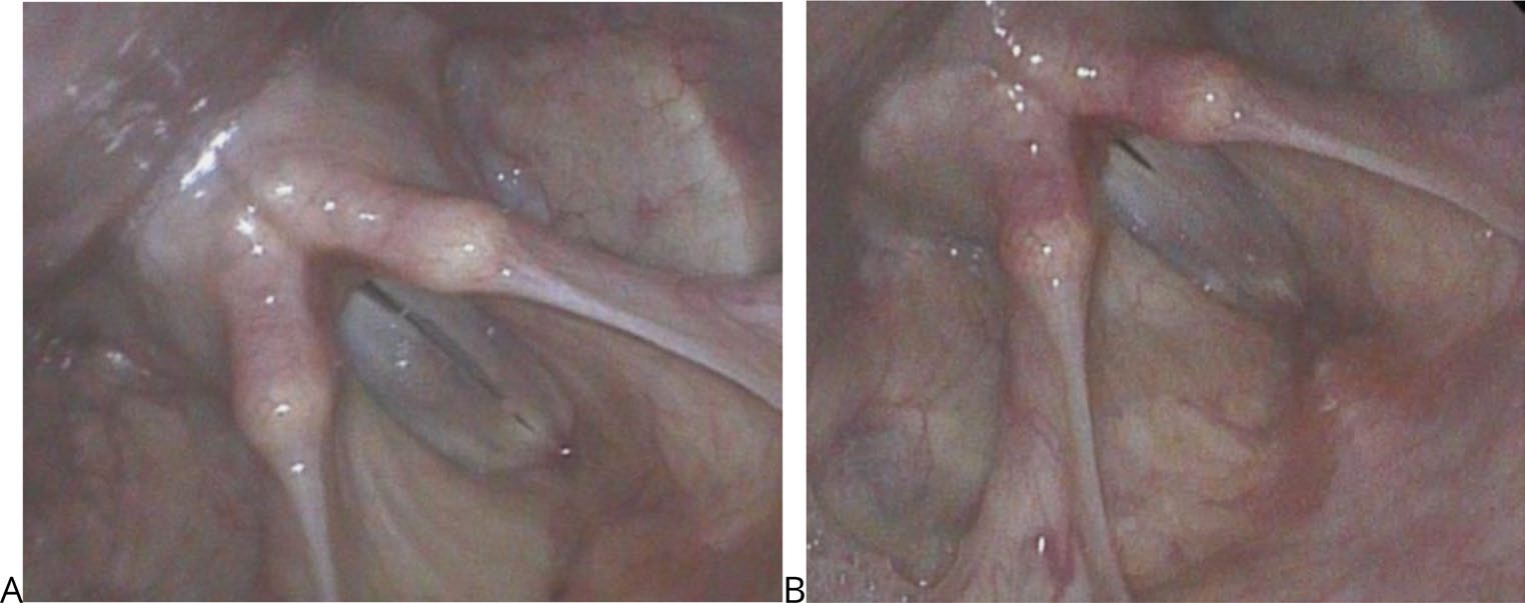
Stroboscopic image of representative case. **A**) Maximal closure position prior to PRP injection demonstrating full length glottal gap and **B**) Maximal closure position 3 months after PRP injection demonstrating complete glottal closure

Changes seen on stroboscopy after PRP injection included improved glottal contact and greater mucosal wave amplitude with smoother free vocal fold margin (Figs. 6 and 7).

### Participant comments

During interviews participants were asked open ended questions around their voice progression such as “How has your voice been?”. Two particular themes were evident where patients consistently reported change – fatigue and effort improvement, and improved vocal consistency.

The following are an example of direct quotes from patients.

Theme 1: Reduction in degree of fatigue or effort. During follow up meetings 20 patients volunteered that they needed less effort to use their voice, or lower levels of fatigue or strain.

E.g “I can sing for one hour, and then I can still speak to my wife when I get home, which I didn’t used to be able to do”.

Theme 2: Improved vocal consistency. During follow up meetings 18 patients volunteered that their voice was either more reliable, or that they had more trust in, or more consistency with their voice.

E.g “I can get through a whole performance knowing my voice won’t break”.

## Discussion

VF pathologies affecting the SLP (VF atrophy, sulcus, scar and inflammatory processes), pose difficulties for treating clinicians [1, 6, 43]. For patient’s reporting symptomatic dysphonia, but who lack pathological changes severe enough to warrant injection of a (temporary) bulking agent via techniques of injection laryngoplasty or Gore-tex™ (permanent) implantation, or for professional voice users for whom pitch alterations related to bulky implants are unpalatable, there has been a lack of alternate therapy available [14, 15, 44]. These patients typically find themselves offered voice therapy and vocal hygiene as their treatment options.

Our study population included a wide variety of dysphonic patients with differing causes for voice change. These range from severe bilateral congenital sulcus, scarring post-squamous cell carcinoma (SCC) resection and radiation, to discreet inflammatory lesions and diffuse chronic laryngitis. Whilst we did treat patients with perceptually severe dysphonia, we also included those with seemingly normal speaking voices, but with symptoms of vocal fatigue after prolonged use, or difficulties in controlling pitch. We were interested to see if PRP could benefit patients at both ends of disease severity and across various pathologies. Previous studies using PRP for benign vocal pathologies have used serial injections in their study protocols. Both Woo [39, 42] and Woerd [38] performed serial injections spaced one month apart, using three and four serial injections respectively. We hypothesised that in some cases only a single injection would be needed for prolonged phonatory benefits and offered participants one initial injection. Serial injections were performed on a patient-by-patient basis depending on response to the first injection. In 26 patients, sustained vocal improvement after a single injection was achieved with no clinical requirement for repeat injections past six months. These participants demonstrated significant improvement in VHI-10 scores at 1,3 and 6 months after injection, compared to baseline. This is the first study to examine single VF PRP injection outcomes in the short and medium term. Whilst further research is needed to assess which patients will see sustained benefits from a single injection, we are encouraged by the potential of a single injection to produce sustained effects. It is possible that patients experiencing repeated VF trauma (i.e. continued phonotrauma, smoking, illness, reflux) may have either greater VF damage or experience ongoing inflammatory mediator presence in the VF and therefore gain short term benefit from an injection or require additional modulation afforded by repeating PRP application [42]. Around half of the participants in this study had received prior surgical treatments for their voice, without achieving satisfactory voice. These treatments included VF injection with hyaluronic acid gels, Gore-tex™ thyroplasty, and laser therapies. PRP injections appeared to assist both closure issues and viscoelastic properties of the SLP, potentially helping voice across more than one realm. Additional study is needed to quantify the degree of benefit in each parameter, and in varying pathologies. These questions pose interesting directions for future research.

Our findings are congruent with other published literature [38, 39, 42], and supports the safety of PRP VF injections, as no significant adverse outcomes occurred during the study period. In our study, office-based PRP injections were well tolerated and 95.7% of patients would consider another injection based upon their experience. Two patients reported discomfort that would preclude them considering office-based treatment, but still noted improvement in phonation and would accept further PRP if under general anaesthesia.

We were able to identify statistical and clinical improvements in a wide range of phonatory outcomes across time points: MPT, VFI, VHI-10 and GRBAS, as previously reported by Woo et al. and Woerd et al., [38, 39, 42]. A 5-point change in VHI-10 score is generally considered to represent clinically significant change in dysphonia associated handicap [45, 46]. In our study 68.1% of patients achieved the MCID for VHI-10 reduction during their follow up period, which is comparable to the results reported in Hirano et al’s 100-person VF bFGF injection study [13]. Our results support the hypothesis that PRP injections can provide phonatory benefit to patients with VF pathology affecting the lamina propria. We observed a consistent patient response trajectory of phonatory improvements reported from 2 to 4 weeks, with additional improvement over 3–6 months. Continued improvement of phonatory parameters over time supports PRP promoting a reparative and regenerative paradigm, rather than providing an immediate medialisation effect such as in traditional injection laryngoplasty [39, 47]. Furthermore, vocal fold appearances on videostroboscopy did not demonstrate persistent fullness as one might expect with bulking agent, and PRP was absorbed rapidly with return to pre-injection voice by the next day.

Given the wide range of subjective dysphonia measurement tools utilized in practice, we chose to measure a selection of different clinical phonatory outcomes in our follow up. Improvement in patient-reported scores matched improved objective measures and clinician-reported tools, suggesting that benefits in perceptual voice quality are evident to the listener, and coupled with improvements in vocal ease for the speaker during phonation. Patients consistently reported reduction in vocal fatigue and increased vocal consistency, regardless of initial vocal deficits which was confirmed by reduction in VFI scores over time. We hypothesise this to be on the basis of two physiological changes in the vocal fold. Firstly, the antifibrotic and inflammatory properties of PRP may be encouraging a physiological shift away from fibrotic processes, improving pliability of VF tissue [24, 28, 30]. Resulting improved propagation of mucosal wave, and reduction in the energy required to commence phonation (phonation threshold pressure) increases glottal contact time and reduces phonatory effort [48]. The changes to mucosal wave propagation seen in our stroboscopic data supports this hypothesis. Secondly, we suspect that the tissue regeneration properties of PRP stimulate some regenerative behaviour in an atrophic vocal fold where functional cells are present but dormant and require a stimulus to reinvigorate them. Stellate cells in the maculae flavae may provide such a pool and be induced to produce SLP elements that improve VF architecture, decreasing glottal insufficiency, and reducing energy expenditure imposed by air loss during phonation through incompletely closed VFs [14, 27, 49–51].

### Limitations

The sample size, and heterogenous sample population are the main limitations of this study, reducing our ability to draw strong conclusions from subgroup analyses. Lack of blinding in rating stroboscopy could be addressed in future studies by using blinded external raters. Limitations in measurement tools may also introduce recall bias and expectation bias but by using a range of patient-based, clinician-based and objective measurements in our study we sought to limit this impact and enhance the strength of our conclusions [52]. In addition, we did not collect tissue specimens for analysis of structural components or molecular change in the VFs, and our PRP preparation technique did not included quantitation of platelet number in the supernatant.

## Conclusions

PRP is an effective and feasible in-office treatment for benign pathology affecting the LP of the VF. Improvements in phonatory measurements following PRP injection were seen across a variety of voice disorders of varying severity (scar, sulcus, atrophy and inflammatory pathology). Improvements were maintained beyond 6 months and in some cases with only a single injection. Further research is needed to determine duration of effects beyond 6 months, and to ascertain which patient populations would benefit sufficiently from a single PRP injection compared to those who are likely to require serial injections.
